# The complete mitochondrial genome of *Dicraeus orientalis* Becker, 1911 (Diptera: Chloropidae)

**DOI:** 10.1080/23802359.2021.1889414

**Published:** 2021-03-16

**Authors:** Jiuzhou Liu, Xin Li, Xiaodong Cai, Bintao Du, Xiaoyan Liu, Ding Yang

**Affiliations:** a Hubei Insect Resources Utilization and Sustainable Pest Management Key Laboratory, College of Plant Science and Technology, Huazhong Agricultural University, Wuhan, China; b College of Plant Protection, China Agricultural University, Beijing, China

**Keywords:** Mitochondrial genome, Carnoidea, Chloropidae, *Dicraeus orientalis*, phylogeny

## Abstract

*Dicraeus orientalis* feeds on the seeds of Poaceae. The complete mitochondrial genome of *D. orientalis* was sequenced and annotated as the first representative of the family Chloropidae. The full length of mitogenome was 16,188 bp, consisting of 13 protein-coding genes (PCGs), 22 transfer RNA genes (tRNAs), and two ribosomal RNA genes (rRNAs). The nucleotide composition was highly A + T biased, accounting for 79.1% of the whole mitogenome. All PCGs start with ATN codons except COI, which end with TAN or incomplete stop codon. ML analysis revealed that Carnoidea was closely related to Ephydroidea and the phylogenetic relationship within Acalyptratae was Tephritoidea + ((Carnoidea + Ephydroidea) + Opomyzoidea).

## Introduction

The Chloropidae, also known as the grass fly, is a large family in the superfamily Carnoidea (Diptera: Acalyptratae) with about 3000 described species worldwide (Nartshuk 2014). They are usually yellow with black or red markings, or mostly to entirely black (Kanmiya 1983; Ismay and Nartshuk 2000). Most species of the family are phytophagous, and some species are important pests of rice and cultivated forage grasses (Nartshuk 2014). Species of Chloropidae regularly occur in grassland habitats (Ismay and Nartshuk 2000).

The specimens of *Dicraeus orientalis* Becker used for this study were collected from Banzhai village (108.04E, 25.24 N), Maolan, Libo, Guizhou by Ding Yang on 13 October 2013 and identified by Xiaoyan Liu. Specimens are deposited in Hubei Insect Resources Utilization and Sustainable Pest Management Key Laboratory in Huazhong Agricultural University, Wuhan, China (Accession number: HZAU0010025). The genomic DNA was extracted from the adult’s whole body (except head) using the DNeasy DNA Extraction kit (TIANGEN) and stored at −20 °C until needed. DNA samples were pooled for next-generation sequencing library construction following Gillett et al. (2014). All quantified DNA extracts were included in a single pool at equimolar concentration, aiming for 50 ng/μL of dsDNA per sample, resulting in a DNA pool of approximately 5 μg. The library was sequenced on an Illumina HiSeq 2500 by BIONONA CO., LTD. Raw read data were filtered and trimmed in Trimmomatic v0.30 (Bolger et al. 2014). The results of high-quality reads (∼4 GB) were used to assemble mitogenomes with the *de novo* assembler IDBA_UD (Peng et al. 2012). The bait sequence COI was amplified by standard PCR reactions, and a BLAST search was carried out with BioEdit 7.0.5.3. The annotations of all tRNA genes were confirmed by ARWEN 1.2 (http://mbio-serv2.mbioekol.lu.se/ARWEN/, Laslett and Canbäck 2008). The complete mitochondrial genome of *D*. *orientalis* (MW368830) was 16,188 bp in length, which consisted of 13 protein-coding genes (PCGs), 22 tRNAs, 2 rRNAs, and control region. The structure of this mitochondrial genome was similar to other Dipteran flies reported previously (Li et al. 2016; Zhou et al. 2017; Qilemoge et al. 2018; Ren et al. 2019). The mitochondrial genome nucleotide composition of *D*. *orientalis* was 41.0% of A, 38.1% of T, 8.7% of G, 12.2% of C, and A + T content was 79.1%. Among the protein-coding genes, six genes use the ATG start codon, five genes use ATT as the start codon, while the cytochrome c oxidase I gene (COI) and NADH-ubiquinone oxidoreductase chain 1 gene (ND1) use TCG and ATA, respectively. The termination codon of these protein-coding genes had four types (9 genes used TAA, 2 genes used TAG, 1 gene used incomplete stop codon TA, and 1 gene used T).

**Figure 1.**
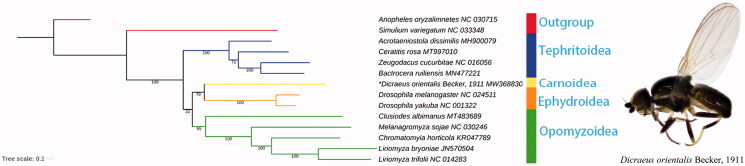
Phylogenetic analysis of 14 Diptera species was generated by using maximum likelihood (ML) method. “*” indicated newly sequenced data in this study.

Based on the nucleotide sequences of 13 PCGs from 14 Diptera species, we conducted a phylogenetic analysis with maximum likelihood (ML) method using RAxML 7.0.3 (Stamatakis 2006). The topology and bootstrap support values were shown in Figure 1. ML analysis revealed that Carnoidea was closely related to Ephydroidea and the phylogenetic relationship within Acalyptratae was Tephritoidea + ((Carnoidea + Ephydroidea) + Opomyzoidea). The complete mitochondrial genome of *D*. *orientalis* could provide valuable information for future studies of Carnoidea phylogeny.

## Data Availability

The data that support the findings of this study are openly available in [NCBI] at [https://www.ncbi.nlm.nih.gov/], reference number [MW368830].
